# Size-Dependent Cytotoxicity of Nanocarbon Blacks

**DOI:** 10.3390/ijms141122529

**Published:** 2013-11-14

**Authors:** Huating Kong, Yu Zhang, Yongjun Li, Zhifen Cui, Kai Xia, Yanhong Sun, Qunfen Zhao, Ying Zhu

**Affiliations:** 1 School of Marine Sciences, Ningbo University, Ningbo 315211, China; E-Mail: kkhhtt88@163.com; 2 Division of Physical Biology, and Bioimaging Center, Shanghai Synchrotron Radiation Facility, Shanghai Institute of Applied Physics, Chinese Academy of Sciences, Shanghai 201800, China; E-Mails: zy20051209@163.com (Y.Z.); changqing871210@163.com (Z.C.); xiakai2012@163.com (K.X.); sunyanhong@sinap.ac.cn (Y.S.); 3 University of Chinese Academy of Sciences, Beijing 100049, China; 4 Key Laboratory of Organofluorine Chemistry and Laboratory of Polymer Materials, Shanghai Institute of Organic Chemistry, Chinese Academy of Sciences, Shanghai 200032, China; E-Mail: liyongjun78@sioc.ac.cn

**Keywords:** nanocarbon blacks (NCBs), size, cytotoxicity, cellular uptake, cytotoxic mechanism

## Abstract

In this study, we investigated the toxic effects of nanocarbon blacks (NCBs) with different sizes to mouse macrophage RAW264.7 cells. MTT and fluorescence-based LIVE assays demonstrated that NCBs uptake caused a size and dose-dependent growth inhibition to the cells. Optical microscopy observations and ^99m^Tc radionuclide labeling techniques were used to investigate the cellular uptake of NCBs with different sizes qualitatively and quantitatively, respectively. Results showed that the cellular uptake amounts of NCBs increased with their increasing size. Large quantities of internal NCBs induced oxidative stress and nuclear damage in cells; these effects may be the critical factors involved in the cytotoxicity of NCBs. The implications associated with these findings are discussed.

## 1. Introduction

Driven by the booming nanotechnology, production and use of nanomaterials are increasing rapidly. Nanomaterials are approaching and entering people’s daily lives due to their massive application. Meanwhile, whether they would adversely affect the environment and human health has become a focus of attention. To date, toxicologists have done a great deal of work to explore the nano-security issue and top scientific journals, including *Science* and *Nature* have published several discussions with serious concerns about nanotoxicology [1,2]. There are about thirty groups (though possibly more, the statistical reports are not complete) that are performing nanotoxicology research, to some extent, in China.

Nanocarbon blacks (NCBs) are well-characterized inert nanoparticles containing a basic carbon nucleus with particle size less than 100 nm and carbon content greater than 99.5%. It is reported that annual carbon black (CB) emissions in China were 1957 Gg in 2007 and increasing in recent years [3]. NCBs are massively manufactured and widely used in industry, for purposes such as colorant of inks, coatings and plastic goods, UV shielding agent of plastic goods and as a reinforcing agent and filler of rubber accounting for 94% of the total carbon black production [4]. In recent years, researchers have carried out a series of *in vivo* and *in vitro* biocompatibility investigations of NCBs. Experimental results in cells show that NCBs can induce DNA damage and strengthen oxidation [5]. In animal models, exposure to NCBs may induce hyperplasia which can lead to pulmonary fibrosis and chemokine expression associated with chronic pneumonia in lung epithelial cells [6]. Among them, the correlation between the particle size and toxicity of NCBs is a hot focus in the study of the security of NCBs. In the work of Koike *et al*., they found that NCBs with mean size of 14 nm have a stronger innate oxidative capacity and induce a more prominent level of oxidative damage in alveolar epithelial cells than NCBs with a diameter of hundreds of nanometers [7]. Similarity, Stone *et al*. reported that 14 nm NCBs stimulated an increase in cytosolic Ca^2+^, and caused a strengthening of significant oxidation while 260 nm NCBs did not result in cytosolic Ca^2+^ increase, oxidative stress or obvious cytotoxicity [8]. However, Magrez *et al*. found that the NCBs with a diameter of hundreds of nanometers caused a more obvious cytotoxicity than multiwalled carbon nanotubes and carbon nanofibers [9]. Until now, the detailed mechanism of cytotoxicity caused by NCBs is still not very clear, and the relationship between cytotoxicity and size of CBs is worthy of further study.

In this work, the mouse macrophage cell line RAW264.7 was chosen as a research model and the toxicity of three types of NCBs with different sizes to cells was systematically compared. The cellular uptake of different sized NCBs was evaluated by optical microscopy and ^99m^Tc radionuclide labeling techniques. The relationship between the size and biological effects of nanoparticles was explored and the toxicity mechanism induced by NCBs was investigated; our results provide important information for nanotoxicity assessment.

## 2. Results

### 2.1. Characterization of NCBs

**Figure 1:**
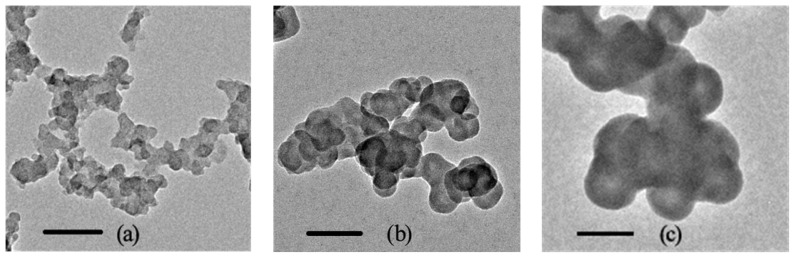
TEM images of NCBs. (**a**) Printex 90; (**b**) Printex G; and (**c**) Flammruss 101. The scale bars correspond to 100 nm.

The manufacturer reports that the aerodynamic diameters are 14, 51 and 95 nm, and the surface areas are 300, 30 and 20 m^2^ g^−1^ for Printex 90, Printex G and Flammruss 101, respectively. The total carbon content of these NCBs is greater than 98 wt %. TEM observations (Figure 1) showed that the size of a single NCB particle was consistent with the information provided by the manufacture. NCBs dispersed in aqueous solution can spontaneously form clusters with a lower free energy. These clusters have a size of tens to hundreds of nanometers (Table S1). The mean particle sizes of NCB clusters in cell culture medium are slightly higher than that in water, probably due to the adsorption of serum proteins in cell culture medium [10]. Fourier transform infrared spectroscopy (FT-IR) showed that these NCBs were functionalized with oxygen-containing groups, e.g., –COOH and –OH (Figure S1), which impart hydrophilicity to NCBs and make them stable in aqueous solution. Zeta potential measurement revealed the surface potential of NCBs in cell culture medium was −17.6, −9.7 and −8.0 mV for Printex 90, Printex G and Flummass 101, respectively (Table S2), illustrating the existence of negatively charged serum proteins on the surface of NCB clusters. Photos of different sized NCB solution at various concentrations also confirm that these nanomaterials were easily dispersed in aqueous solution and complete cell culture medium by sonication (Figure S2).

### 2.2. Cytotoxicity Determination of NCBs

**Figure 2:**
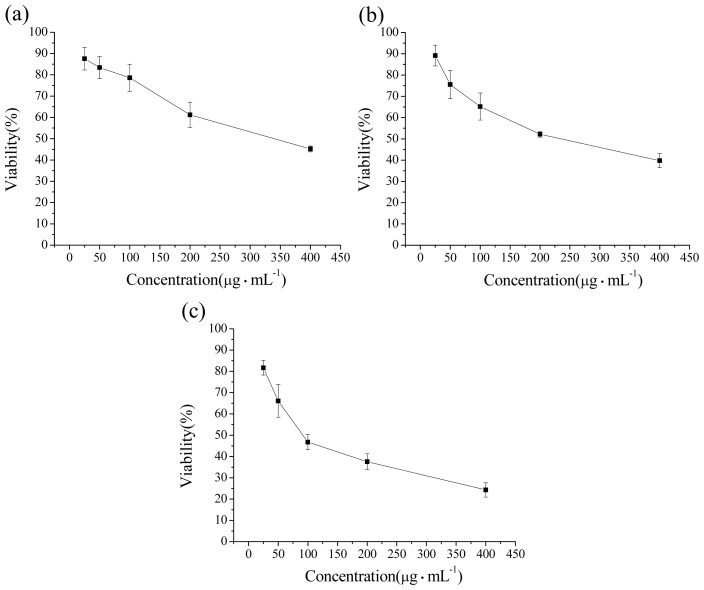
Viability determination of RAW264.7 cells exposed to different sized NCBs for 24 h. (**a**) Printex 90; (**b**) Printex G; and (**c**) Flammruss 101.

We first determined the cytotoxic effect of the three types of NCBs (Figure 2) by MTT methods. Results showed that after exposure for 24 h, the number of viable cells exposed to 14 and 51 nm NCBs did not show any difference with the control groups at a low concentration of 25 μg mL^−1^. When the concentration was higher than 50 μg·mL^−1^, all of these NCBs showed cytotoxic effects, and cell viability decreased with increasing concentration of NCBs, displaying obvious concentration-dependent effects. Moreover, we observed the cell viability decreased with the increasing size of NCBs at each exposure concentration. For example, at concentration of 400 μg·mL^−1^, the cell viabilities were 45%, 39% and 24% after 14, 51 and 95 nm NCBs treatment, respectively.

**Figure 3:**
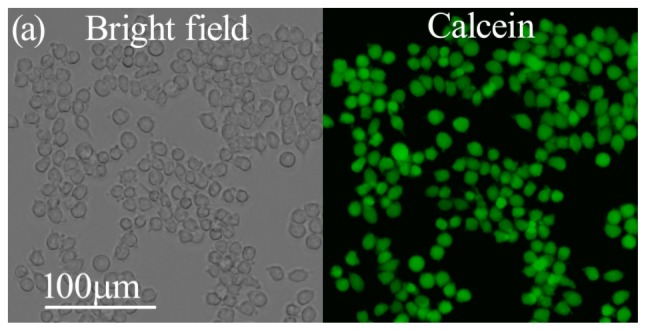

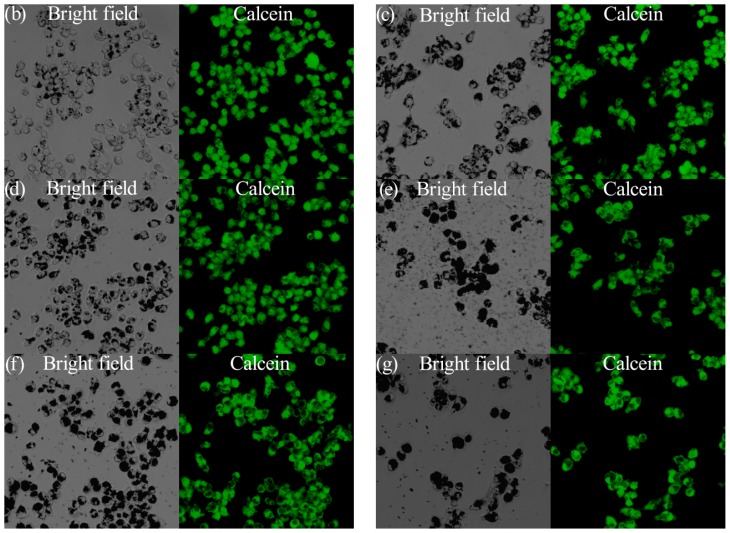
The live stains for RAW264.7 cells after incubation with different sized NCBs for 24 h. (**a**) Control cells; (**b**,**c**) Cells after incubation with (**b**) 50 μg·mL^−1^ and (**c**) 200 μg·mL^−1^ Printex 90; (**d**,**e**) Cells after incubation with (**d**) 50 μg·mL^−1^ and (**e**) 200 μg·mL^−1^ Printex G; (**f**,**g**) Cells after incubation with (**f**) 50 μg·mL^−1^ and (**g**) 200 μg·mL^−1^ Flammruss 101. The scale bar corresponds to 100 μm.

Calcein-AM is a type of cell-permeant probe that can be used to determine cell viability in most eukaryotic cells. The nonfluorescent calcein-AM is converted to green-fluorescent calcein in living cells, after acetoxymethyl ester hydrolysis by intracellular esterases. Calcein-AM staining is widely used to assess the cytotoxicity of nanoparticles [11–13]. Further calcein-AM staining results are in agreement with the determination obtained from the MTT assays (Figure 3).

### 2.3. Uptake of NCBs in RAW264.7 Cells

**Figure 4:**
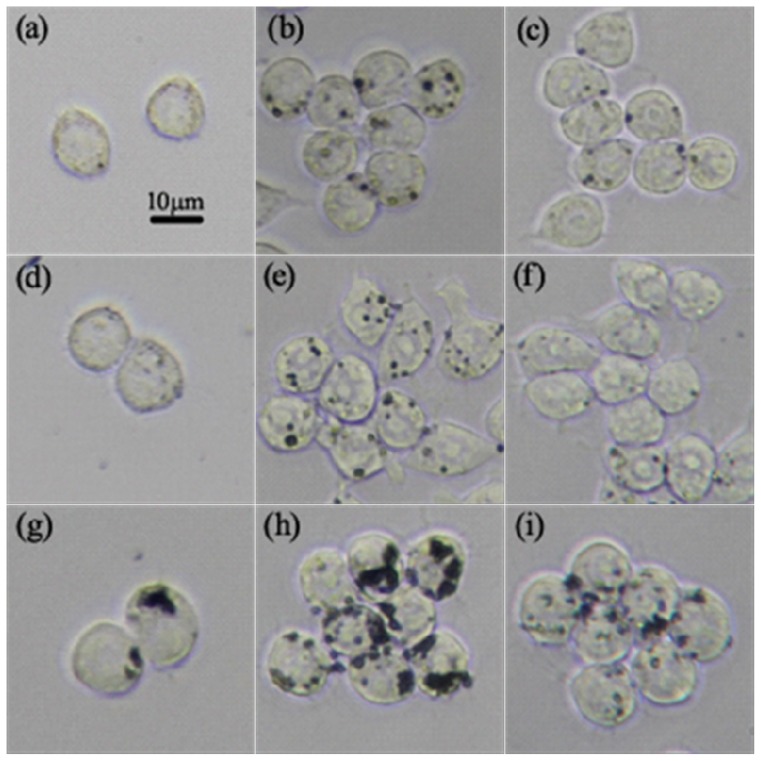
Optical microscope images of RAW264.7 cells after incubation with 50 μg·mL^−1^ different sized NCBs. (**a**,**d**,**g**) Cells after 2 h incubation with NCBs; (**b**,**e**,**h**) Cells after 24 h incubation with NCBs; (**c**,**f**,**i**) Cells after 48 h incubation with NCBs; (**a**–**c**) Printex 90; (**d**–**f**) Printex G; (**g**–**i**) Flammruss 101. The scale bar corresponds to 10 μm.

Uptake of NCBs in RAW264.7 cells was then analyzed by optical microscopy. After exposed to 14 and 51 nm NCBs for 2 h, images showed that there were small aggregates with light color inside cells (Figure 4a,b), while after treatment with 95 nm NCBs for 2 h, there were dark aggregates inside cells (Figure 4c), indicating the cellular uptake amount of 95 nm NCBs was far more than that of the other two NCBs. After 24 h incubation, the black aggregates inside the cells increased significantly (Figure 4d–f), showing obvious cell uptake of NCBs over time. However, after 48 h incubation, we clearly observed a notable decrease in intracellular black aggregates (Figure 4g–i), suggesting exclusion of intracellular NCBs by exocytosis [14]. In addition, after 24 and 48 h incubation, the cell uptake amount of 95 nm NCBs was also much higher than that of the other two NCBs (Figure 4).

Radionuclide labeling and tracing techniques are used to quantitatively determine the location and distribution of nanoparticles in living systems. Recently, various radionuclides such as ^99m^Tc, ^67^Ga, ^125^I, ^64^Cu and ^111^In have been successfully used to track nanoparticles and conduct studies on their bioeffects [15–19]. In this work, the cellular uptake of NCBs with different diameters was measured quantitatively by the ^99m^Tc radiolabeling technique. Paper chromatography analysis showed the labeling yields of 14, 51 and 95 nm NCBs were 92%, 95% and 94%, respectively (Figure S3). The radiochemical purities of the labeling NCBs in water and cell culture medium were examined after simple purification with centrifugation and wash. As shown in Figure S4, a small amount of radionuclide ^99m^Tc released from the ^99m^Tc-labeling NCBs over time. The released amount of radionuclide in complete cell culture medium was higher than that in water, probably due to the competitive adsorption between components in medium and radionuclide ^99m^Tc on NCBs [20]. The radiochemical purity of ^99m^Tc-NCBs maintained a fairly good level of nearly 75% in complete cell culture medium after 48 h incubation, thus the ^99m^Tc-labeling NCBs were able to be used for experimental study of intracellular uptake.

After incubation with the cells for 2 h, the radioactivity measurements showed the uptake amounts of 95 nm NCBs was 52 ng/1000 cells, which is much higher than that of the other two NCBs. At 24 h, the uptake amounts of 14, 51 and 95 nm NCBs were 32, 40 and 80 ng/1000 cells, respectively, indicating the cellular uptake of NCBs increased with the elapsed time. However, after 48 h incubation, the cellular uptake of three kinds of NCBs decreased to different extents (Table 1). These radionuclide-tracing results are in good agreement with the optical microscopy observation.

### 2.4. Mechanisms of NCBs-Induced Cytotoxicity

**Figure 5:**
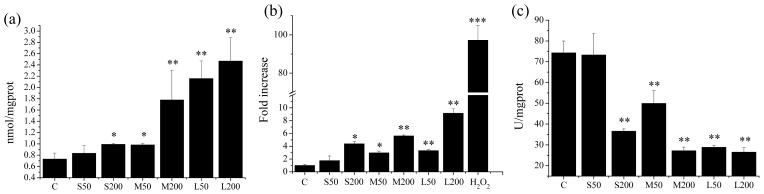
NCBs-induced oxidative stress in RAW264.7 cells. (**a**) MDA level; (**b**) ROS level; (**c**) SOD level. C: control; S50: 50 μg·mL^−1^ Printex 90; S200: 200 μg·mL^−1^ Printex 90; M50: 50 μg·mL^−1^ Printex G; M200: 200 μg·mL^−1^ Printex G; L50: 50 μg·mL^−1^ Flammruss 101; L200: 200 μg·mL^−1^ Flammruss 101. * *p* < 0.05; ** *p* < 0.01; *** *p* < 0.001; one-way ANOVA for comparison.

Several studies have shown that oxidative damage is one of the important causes of cytotoxicity of nanoparticles [21,22]. MDA (malondialdehude), a major metabolite of lipid peroxidation, is a marker of oxidative stress in cells. ROS (reactive oxygen species) are chemically reactive molecules containing oxygen. ROS associated oxidative stress is the general pathway for nanoparticle to induce cell toxicity [23,24]. SOD (superoxide dismutase) is a key enzyme of antioxidation *in vivo* that clears superoxide anion radicals in the oxidation process; a decline in SOD activity results in the accumulation of superoxide anion radicals, causing cell impairment through lipid peroxidation of cell membranes, which leads to the destruction of normal physiological function of the cells [25]. Therefore, we measured the MDA, ROS and SOD levels in cells after incubation with different sized NCBs to determine whether oxidative damage occurred. Results showed that compared to the control groups, 14 nm NCBs did not induce any increase of MDA level in the cells at concentration of 50 μg·mL^−1^ and caused a slight rise of MDA level at concentration of 200 μg·mL^−1^. The MDA level of cells induced by 51 nm NCBs increased with increasing concentration. A significant increase of MDA level in the cells was detected after treatment with 95 nm NCBs, regardless of their concentration (Figure 5a). The intracellular ROS level after exposure to different sized NCBs had similar trends (Figure 5b). Correspondingly, 14 nm NCBs did not induce a decrease of SOD activity in the cells at a concentration of 50 μg·mL^−1^ though caused a slight decline of SOD activity at a concentration of 200 μg·mL^−1^. The SOD activity of cells was significantly inhibited by the other two NCBs (Figure 5c).

**Figure 6:**
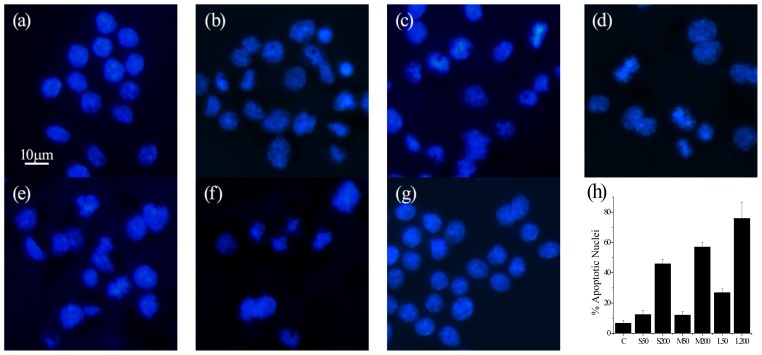
NCBs-induced nuclear damage in RAW264.7 cells. (**a**) 50 μg·mL^−1^ Printex 90; (**b**) 200 μg·mL^−1^ Printex 90; (**c**) 50 μg·mL^−1^ Printex G; (**d**) 200 μg·mL^−1^ Printex G; (**e**) 50 μg·mL^−1^ Flammruss 101; (**f**) 200 μg·mL^−1^ Flammruss 101; (**g**) Control. The scale bar corresponds to 10 μm. (**h**) Quantitative calculation of the percent of apoptotic nucleic after treatment with different sized NCBs at various concentrations. C: control; S50: 50 μg·mL^−1^ Printex 90; S200: 200 μg·mL^−1^ Printex 90; M50: 50 μg·mL^−1^ Printex G; M200: 200 μg·mL^−1^ Printex G; L50: 50 μg·mL^−1^ Flammruss 101; L200: 200 μg·mL^−1^ Flammruss 101.

Nuclear damage is another important mechanism by which nanoparticles induce cytotoxicity [26–28]. In this work, after treatment with NCBs, cell nuclei were stained with Hoechst 33258. Morphological changes of cell nuclei were observed under a fluorescence microscope. As shown in Figure 6, cell nuclei in control groups showed homogeneously stained nuclei. 50 μg·mL^−1^ 14 nm NCBs did not induce obvious morphological changes of the cell nucleus, while 200 μg·mL^−1^ induced partial shrinkage and nuclei deformation, implying some nuclear damage. In the case of the other two NCBs with larger size, 50 μg·mL^−1^ nanoparticles induced slight nuclear damage and 200 μg·mL^−1^ nanoparticles resulted in serious nuclear damage such as dramatic shrinkage and deformation with chromatin aggregation. Nuclei that had undergone fragmentation, chromatin marginalisation and condensation were identified as cells that were most likely to be apoptotic and quantitative calculation of the percent of apoptotic nucleic after treatment with different sized NCBs at various concentration is given in Figure 6h.

## 3. Discussion

With the increasing application of NCBs, the impacts of NCBs on the environment and human health have attracted the attention of researchers. A large number of studies have shown that NCBs with different particle sizes are able to cause a range of biological toxicity including varying degrees of oxidative stress, gene mutation and inflammation [29–32].

In consideration of the different sizes of NCBs, one of the priorities of toxicity study is the correlation between the particle sizes and toxic effects of NCBs. It has been shown that compared with the micro-sized CB, nano-sized CB produced more severe inflammation and oxidative DNA damage [33]. Hussain *et al*. [34] investigated the capacity of NCBs (with diverse particle diameter and specific surface areas) to induce cellular inflammatory effects and promote oxidative stress. Results showed that the smaller NCBs with larger specific surface areas produced a greater ability to promote oxidation and proinflammatory effects, and resulted in higher cytotoxicity. In the study of Rim *et al*. [35], proinflammatory effects and DNA damage induced by NCBs with diameters of 30 and 200 nm in mouse macrophages were compared and they found that 30 nm NCBs produced more proinflammatory cytokine expression and DNA fragmentation, which led to higher cytotoxicity than that caused by the 200 nm NCBs. In the present study, using RAW264.7 cells, we investigated the interactions between NCBs with diameters of 14, 51 and 95 nm. MTT and fluorescence-based LIVE assays, demonstrated that NCBs-induced cytotoxicity was size-dependent; optical microscopy observations and radionuclide tracing results showed that NCBs could be efficiently internalized by cells, regardless of particle size, and that the NCBs with larger diameter were more conducive to internalization by cells. In combination with cytotoxicity determination of NCBs, it was found that the cytotoxicity of NCBs increases with their increasing size, and that there is a positive correlation between the cellular uptake of NCBs and their cytotoxicity. SOD, ROS and MDA levels in cells, as well as cell staining results, showed that NCBs inside cells induced concentration and size-dependent oxidative stress and nuclear damage, which may be critical factors for the cytotoxicity of NCBs. Similar, Chithrani *et al*. [36] investigated the size dependence of gold nanoparticles in Hela cells. Results showed that 50 nm gold nanoparticles entered cells more efficiently than smaller nanoparticles. Recently, Ma *et al*. [37] reported that the uptake of gold nanoparticles in normal rat kidney (NRK) cells was also size-dependent, with larger gold nanoparticles more readily taken up by the cells. Gold nanoparticles that entered the cells were transported to the lysosomal compartment and the larger gold nanoparticles caused more serious impairment of lysosomal function.

From the evidence mentioned above, we believe that there is a close relationship between cytotoxicity and particle size of NCBs. The nano-sized CBs with diameter in aqueous solution below 500 nm can be internalized by cells through phagocytosis, clathrin-mediated endocytosis, caveolin-mediated endocytosis and other pathways [38]. In this size range, the cellular uptake of NCBs increases with increasing size of NCBs, which leads to increasing cytotoxicity. However, CBs with diameters in aqueous solution greater than 500 nm, or even up to micron-level, may not be taken up by cells effectively through clathrin-mediated endocytosis or by caveolin-mediated endocytosis pathways. Only a small amount of them can enter into cells by macropinocytosis pathway or even cannot enter into cells due to the micrometer size [39]. Therefore, the cytotoxicity of micro-sized CBs is likely to be lower than that of nano-sized CBs. In the present work, we measured the toxicity of micro-sized carbon particles (Sigma, Shanghai, China) (Figure 1d, Tables S1 and S2, and Figure S5) for RAW264.7 cells by MTT assay. Results showed CBs at various concentration showed no obvious toxicity to cells after 24 h incubation (Figure S6).

As researchers begin to construct nanostructures, issues of size will be important. In this work, we demonstrated the cellular uptake and cytotoxicity of NCBs was heavily dependent upon size. NCBs with larger particle size were more easily taken up by cells, induced higher oxidative stress and more serious nuclear damage, which then led to more severe toxic effects to cells. All of these results will have implications in the design of nanostructures for daily application.

## 4. Experimental Section

### 4.1. Characterization of NCBs

Three types of NCBs (Printex 90, Printex G, Flammruss 101) were obtained from Degussa (Shanghai, China). The mean aerodynamic diameters are 14, 51 and 95 nm for Printex 90, Printex G and Flammruss 101, respectively. The NCBs were suspended in distilled water to reach the concentration of 2 mg·mL^−1^. The stock suspensions were stored at 4 °C and ultrasonicated for 20 min before use. The average primary particle sizes and aggregation levels of NCBs dispersed in the water were confirmed by transmission electron microscopy (TEM, FEI, Tecnai G^2^ F20, Hiilsboro, OR, USA) analysis. The concentration of the samples used for TEM was 10 μg·mL^−1^. The hydrated diameters of the NCBs distributed in water and cell culture medium were measured by dynamic light scattering (DLS) using a Beckman Coulter nanoC particle analyzer (Beckman Coulter, Inc., Brea, CA, USA). The zeta potential determination of NCBs in cell culture medium was performed on a Beckman Coulter nanoC particle analyzer. Fourier transform-infrared spectroscopy (FT-IR) was completed by a Nicolet AVATAR-360 FT-IR spectrophotometer (Thermo Nicolet Corp. Madison, WI, USA) to show the functional groups on the surface of NCBs.

### 4.2. Cytotoxicity Assessments of NCBs

RAW264.7 macrophage-like cells, purchased from the Type Culture Collection of the Chinese Academy of Sciences, were cultured (37 °C, 5% CO_2_) in DMEM (Dulbecco’s modified Eagle’s medium, Gibco, Grand Island, NY, USA) medium supplemented with 10% fetal bovine serum (FBS), 4.5 mg·mL^−1^ glucose, 1.5 mg·mL^−1^ NaHCO_3_, 100 U·mL^−1^ streptomycin, 100 μg·mL^−1^ penicillin and 4 mM l-Glutamine.

A cell suspension (10^5^ cells/well) was dispensed into 24-well plates and incubated overnight to allow for cell adherence. After washing twice with phosphate buffered saline (PBS), cells were treated with different sized NCBs at various concentration (25, 50, 100, 200 and 400 μg·mL^−1^). Cells without NCBs were used as controls. After 2, 24, and 48 h incubation, the cell viability was determined by the [3-(4,5-dimethylthiazol-2-yl)-2,5-diphenyltetrazolium bromide] MTT assay (Sigma-Aldrich, Shanghai, China) and expressed as a percentage of OD_treat_/OD_control_. All viability determination data were based on three independent experiments.

Fluorescence-based LIVE assay was used to distinguish the live cells. Cells for imaging were seeded on 24-well culture plates at a density of 5 × 10^4^ cells/well and incubated overnight to allow for cell adherence. After exposure to different sized NCBs at concentration of 50 and 200 μg·mL^−1^ for 24 h, the cells were washed thrice with PBS and stained with calcein AM (Invitrogen, Carlsbad, CA, USA, 2 μM, 10 min, Ex/Em: 494 nm/517 nm). Excess calcein-AM was washed with PBS and all samples were observed with the corresponding filters under a Leica DMI3000 B fluorescent microscope (Leica, Solms, Germany).

### 4.3. Observation for Cellular Uptake of NCBs by Optical Microscopy

RAW264.7 cells were seeded on 24-well culture plates at a density of 5 × 10^4^ cells/well. After exposure to different sized NCBs at the concentration of 50 μg·mL^−1^ for various time (2, 24 and 48 h), the cells were washed with PBS and all samples were observed under an Olympus CKX41 optical microscope (Olympus, Tokyo, Japan).

### 4.4. ^99m^Tc Labeling of NCBs

Three types of NCBs were labeled with ^99m^Tc by the same protocol as previously reported [10]. In brief, NCBs were dispersed in millipore water with an ultrasonic device for 5 min, and then ascorbic acid and stannous chloride were added. The solution was adjusted to pH 6–7, and then radiotracer Na^99m^TcO_4_ was added to the suspension. The mixture was stirred at 40 °C for 5 min. After centrifugation, the unreacted Na^99m^TcO_4_ was removed through more than five washing/centrifugation cycles. Paper chromatography (PC, Hangzhou Xinhua Paper Co., Ltd., Hangzhou, China) analysis developed with 0.9% normal saline was used to determine the labeling yields and for evaluation of chemical stability of the labeling compounds.

### 4.5. Measurement of Cellular Uptake of NCBs with Radiotracer Techniques

NCBs labeled with ^99m^Tc were dispersed in cell culture medium at concentration of 2 mg·mL^−1^. RAW264.7 cells were seeded on 24-well culture plates at a density of 10^5^ cells/well. Cells were then incubated with three types of NCBs at the final concentration of 50 μg·mL^−1^ for 24 h. The medium without NCBs was used as control. After a PBS wash, cells were harvested with 0.25% EDTA-trypsin, and suspended in 1.05 mL cell culture medium. 50 μL of the cell suspension was used to determine the number of cells and the remaining cells was collected to determine the activity of NCBs labeled with ^99m^Tc inside cells. 100 μL of the stock NCBs solutions was set as standard sample and its radioactivity was counted to measure the total radioactivity of the NCBs labeled with ^99m^Tc in each well. The uptake amounts of NCBs per 1000 cells (ng/1000 cells) were calculated by

(1) $$\text{Uptake amounts}/1000\;\text{cells}=\frac{R_{c}\times 200\times 1.05}{R_{s}\times 10\times n}$$

where *R*_c_ is the radioactivity of the cell suspension, *R*_s_ is the radioactivity of the standard samples, and *n* is the number of cells, respectively.

### 4.6. Determination of SOD Reactivity and MDA Production Induced by NCBs

RAW264.7 cells were seeded on 60 mm dishes at a density of 10^6^ cells/well. Three types of NCBs were added to cell cultures with the final concentration of 50 and 200 μg·mL^−1^, respectively. After 24 h incubation, cells were washed thrice with PBS, and then sonicated in ice-cold PBS for 5 min. The homogenate was centrifuged for 15 min at 14,000 rpm at 4 °C, and the supernatants were used to measure the levels of SOD and MDA. The SOD and MDA values in cells are assessed using Detection Kits for SOD and MDA (Jiancheng, Nanjing, China).

### 4.7. Measurement of Intracellualr ROS Production

Cells were treated with different sized NCBs as mentioned above. Cells treated with 100 mM H_2_O_2_ for 15 min were used as positive control. After 24 h incubation, cells were washed with PBS and loaded with dichlorofluorescin diacetate (DCFH-DA, 5 μM) from Reactive Oxygen Species Assay Kit (Beyotime Institute of Biotechnology, Jiangsu, China) for 40 min. After a further wash with PBS, cell suspensions were collected into 96-well flat bottom black plate to determine the relative fluorescent intensity (RFI, λ_ex_ 488 nm, λ_em_ 525 nm) by Biotek Synergy H1 hybrid reader. The cell culture medium was used as blank. The RFI over control was calculated as the measured ROS levels.

### 4.8. Observation of Nuclear Damage Induced by NCBs

Cells for imaging were grown on 24-well plates with cover slips. After exposure to different sized NCBs at concentration of 50 μg·mL^−1^ and 200 μg·mL^−1^ for 24 h, the cells were washed thrice with PBS and then fixed with 4% paraformaldehyde-4% sucrose for 30 min. Following a further wash with PBS, cell nuclei were stained with DNA-specific fluorescent Hoechst 33258 (Sigma, Shanghai, China, 5 μg·mL^−1^, 10 min, *E*_x_/*E*_m_: 352 nm/461 nm). All samples were observed under a Zeiss Axioskop2 plus fluorescent microscope (Carl Zeiss, Gottingen, Germany). A total of 100 cells per sample were analyzed and the percentage of the percent of apoptotic nucleic after treatment with different sized NCBs at various concentrations was calculated.

## 5. Conclusions

In this study, the interactions between RAW264.7 cells and three types of NCBs with particle sizes of 14, 51 and 95 nm were investigated. Results showed that the quantity of NCBs cellular uptake increased with the increasing size of the particles, and increasing uptake quantity induced a corresponding increase in oxidative stress and nuclear damage, processes which may be critical factors involved in the cytotoxicity of NCBs. The results presented here thus provide important data for toxicology studies of NCBs.

## Supplementary Information



## Figures and Tables

**Table 1:** Cellular uptake amounts of ^99m^Tc-NCBs (ng/1000 cell, mean ± SD).

Incubated time/h	14 nm NCBs	51 nm NCBs	95 nm NCBs
2	10.2 ± 2.2	13.4 ± 1.9	51.9 ± 3.8
24	32.3 ± 3.2	39.7 ± 2.9	78.4 ± 2.8
48	24.5 ± 3.4	31.4 ± 4.1	58.6 ± 3.4
