# Supplementary material for: Size-Dependent Cytotoxicity of Nanocarbon Blacks
Source: Int J Mol Sci. 2013 Nov 14;14(11):22529–43. doi: 10.3390/ijms141122529 (PMC3856077; doi:10.3390/ijms141122529)
Supplement: Supplementary file 1 [file ijms-14-22529-s001.pdf]

Supplementary Information

Figure S1. FT-IR analysis of NCBs and micro-sized CBs. (**a**) Printex 90; (**b**) Printex G; (**c**) Flummass 101; (**d**) Micro-sized CBs.

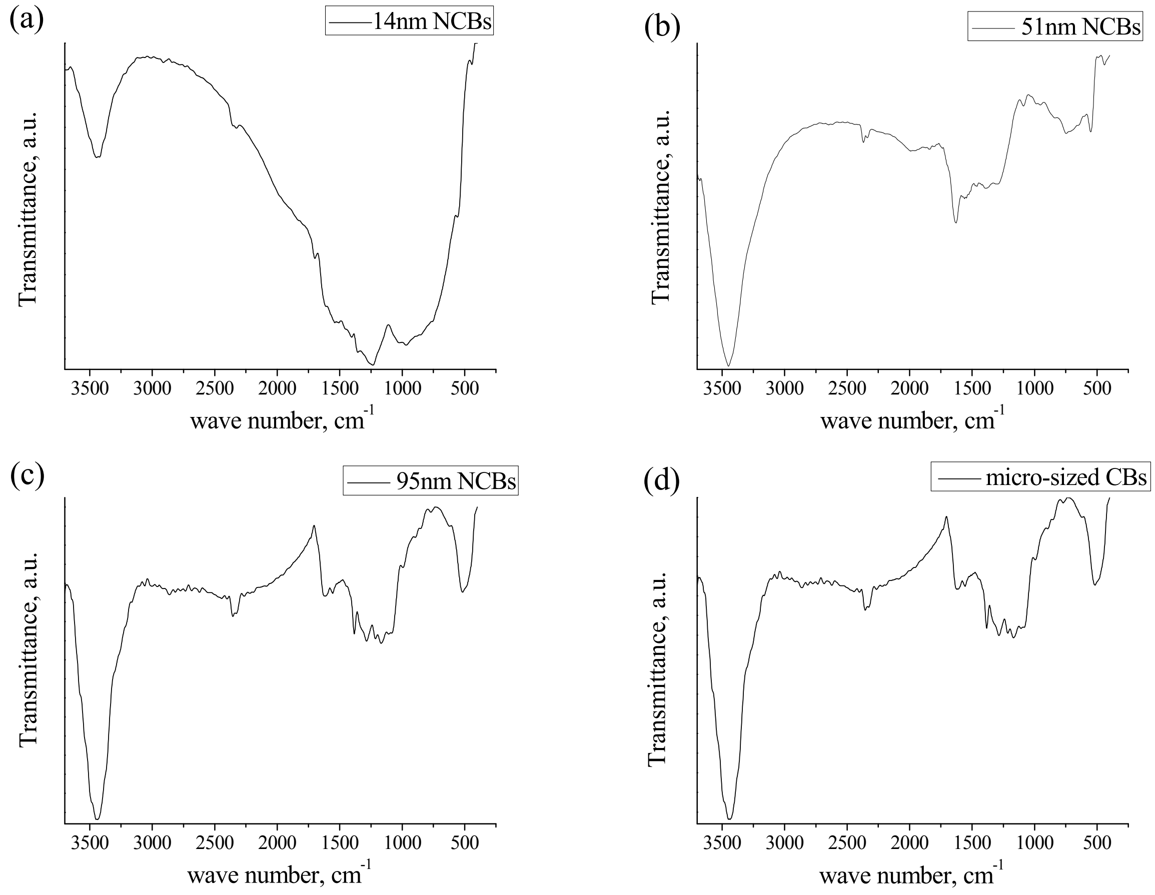

Figure S2. Photos of different sized NCB solution at various concentration. (**a**) 50 μg·mL^{-1}; (**b**) 200 μg·mL^{-1}.

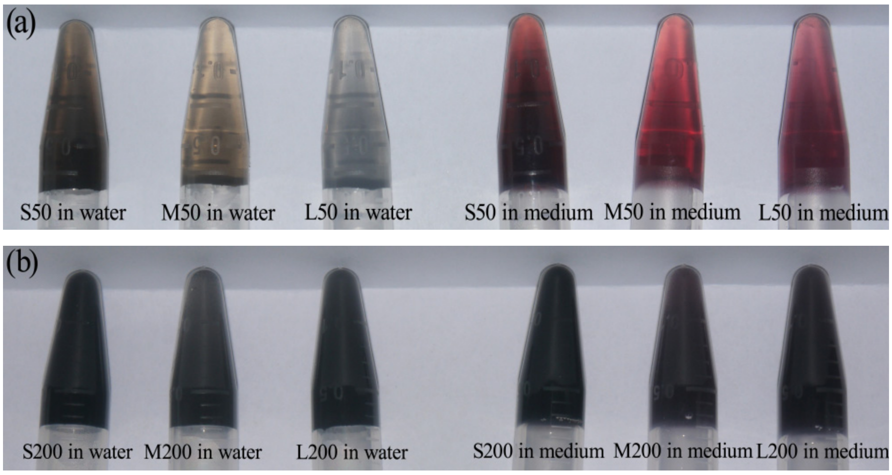

Table S1. The size distribution of NCBs and micro-sized CBs determined by DLS.

**CBs**	**Mean partical size(nm)**	
Dispersion medium	Water	Medium
14 nm NCBs	95.1	102.2
51 nm NCBs	158.8	176.4
95 nm NCBs	355.2	403.9
micro-sized CBs	2731.8	2917.8

Table S2. The zeta potential of NCBs and micro-sized CBs in cell culture medium.

**CBs**	**Mean zeta potential (mV)**
14 nm NCBs	−17.6
51 nm NCBs	−9.7
95 nm NCBs	−8.0
micro-sized CBs	−7.4

Figure S3. The radiochemical purity of ^{99m}Tc-NCBs after purification. (**a**) Printex 90; (**b**) Printex G; (**c**) Flummass 101.

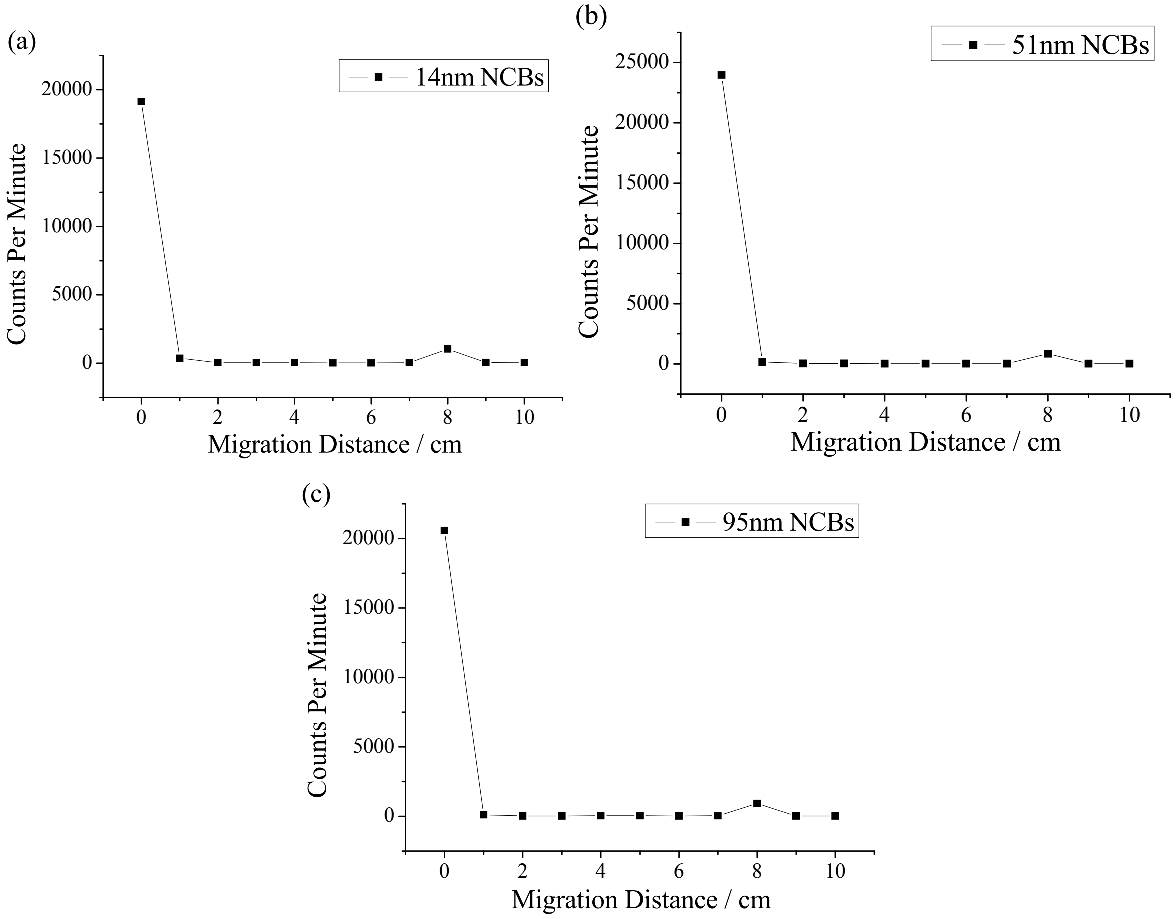

Figure S4. The radiochemical purity of ^{99m}Tc-NCBs in millipore water and cell culture medium at the time points of 1, 2, 4, 24 and 48 h. (**a**) Printex 90; (**b**) Printex G; (**c**) Flummass 101.

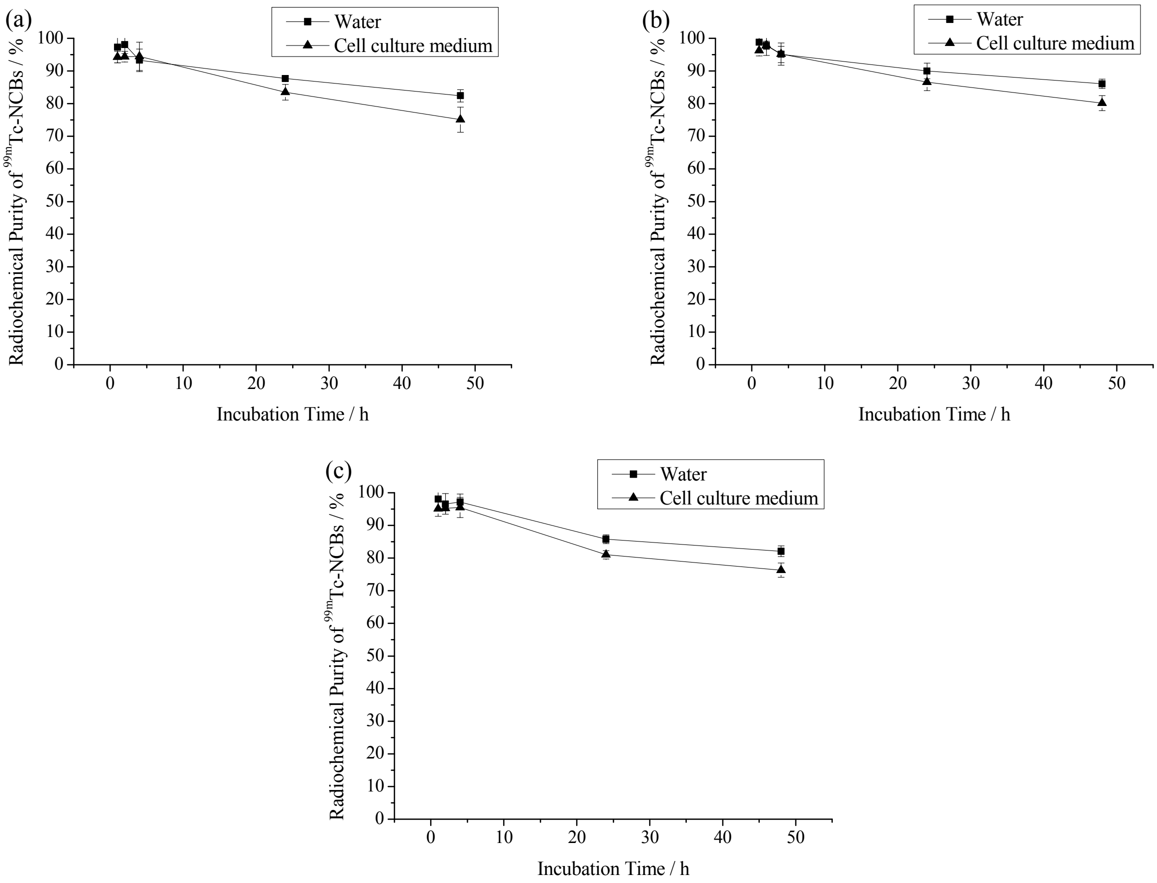

Figure S5. TEM image of micro-sized CBs.

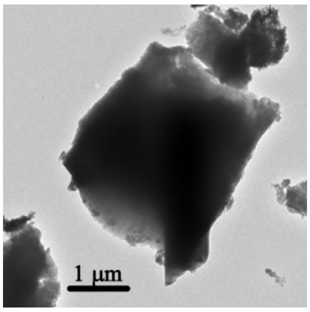

Figure S6. Viability determination of RAW264.7 cells exposed to micro-sized CBs for 24 h.

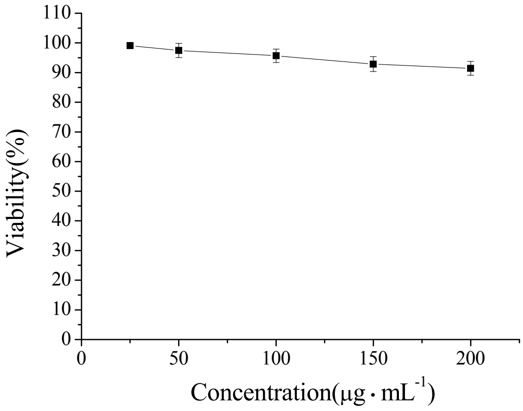

© 2013 by the authors; licensee MDPI, Basel, Switzerland. This article is an open access article distributed under the terms and conditions of the Creative Commons Attribution license (http://creativecommons.org/licenses/by/3.0/).
